# On total turbulent energy and the passive and active role of buoyancy in turbulent momentum and mass transfer

**DOI:** 10.1007/s10236-012-0536-6

**Published:** 2012-04-29

**Authors:** Michel A. J. de Nijs, Julie D. Pietrzak

**Affiliations:** 1 Environmental Fluid Mechanics Section, Delft University of Technology, PO Box 5048, Stevinweg 1, 2600 Delft, The Netherlands; 2 Van Oord Dredging and Marine Contractors b.v., Estimating and Engineering department, Rotterdam, The Netherlands

**Keywords:** Stratified shear flow, Total turbulent energy, Vertical turbulent kinetic energy, Available turbulent potential energy, Countergradient buoyancy fluxes, Turbulent Prandtl number, Energetic turbulent structures, Convective motions

## Abstract

Measurements of turbulent fluctuations of horizontal and vertical components of velocity, salinity and suspended particulate matter are presented. Turbulent Prandtl numbers are found to increase with stratification and to become larger than 1. Consequently, the vertical turbulent mass transport is suppressed by buoyancy forces, before the turbulent kinetic energy (TKE) and vertical turbulent momentum exchange are inhibited. With increasing stratification, the buoyancy fluxes do not cease, instead they become countergradient. We find that buoyantly driven motions play an active role in the transfer of mass. This is in agreement with trends derived from Monin–Obukhov scaling. For positive Richardson flux numbers (Ri_*f*_), the log velocity profile in the near-bed layer requires correction with a drag reduction. For negative Ri_*f*_, the log velocity profile should be corrected with a drag increase, with increasing |Ri_*f*_|. This highlights the active role played by buoyancy in momentum transfer and the production of TKE. However, the data do not appear to entirely follow Monin–Obukhov scaling. This is consistent with the notion that the turbulence field is not in equilibrium. The large stratification results in the decay of turbulence and countergradient buoyancy fluxes act to restore equilibrium in the energy budget. This implies that there is a finite adjustment timescale of the turbulence field to changes in velocity shear and density stratification. The energy transfers associated with the source and sink function of the buoyancy flux can be modeled with the concept of total turbulent energy.

## Introduction

Boundary layer shear flows dominate the atmosphere, oceans, estuaries, and lakes. It is well known that such boundary layers are dominated by energetic turbulent structures, Kline et al. 1967; Smith 1984; Nezu and Nakagawa 1993; Adrian et al. 2000; Natrajan and Christensen 2006. This 3D turbulence is characterized by a quasicyclic process of ejection and sweep events. These intermittent motions are bounded at the small scales by molecular viscosity and at the large scales by the characteristic length scales of the domain. In stratified shear flows, the scales are also inhibited by buoyancy forces. In stratified flows, intermittent motions also include convective motions and internal gravity waves (Turner 1973). These intermittent motions cause both downgradient and countergradient transports.

Based on large eddy simulation (LES) results, Gerz and Schumann (1996) developed conceptual models for countergradient transport of momentum and heat by intermittent motions in stratified homogeneous shear flows. These principles also hold for nonhomogeneous turbulent conditions, (de Nijs and Pietrzak 2011). Based on quadrant and spectral analysis, they determined that at relatively large stratification and shear, the turbulent transports of momentum and mass are governed by energetic turbulent structures produced by velocity shear, while at large stratification and weak shear remnants of transports by energetic turbulent structures in the past induce convective motions. These convective motions begin to actively determine the turbulent structure and hence the transport of momentum and mass. However, it is not feasible to resolve all these motions in numerical models at the scales relevant for geophysical flows.

Traditionally, Reynolds-averaged Navier–Stokes (RANS) modeling has been used to simulate environmental and geophysical flows. The Mellor–Yamada level 2 models (Mellor and Yamada 1974), *k*–ε (Launder and Sharma 1974; Rodi 1984) and *k*-ω (Wilcox 1988; Umlauf et al. 2003) turbulence closure models are widely used examples. The influence of buoyancy forces is typically taken into account as a sink of turbulent kinetic energy (TKE), in the case of stable stratification (e.g., Burchard et al. 1998, 2008) which reduces the value of the eddy viscosity compared to neutral conditions. These models make the assumption that turbulence is downgradient. Turbulence closure, including countergradient transport for RANS models, is the subject of this study.

In some RANS models, diffusivities for salinity and suspended particulate matter (SPM) are taken proportional to turbulent viscosity through a constant turbulent Prandtl number (σ_*T*_) of about 1. However, Ellison (1957) derived an expression for σ_*T*_ and suggested that the vertical turbulent transport of heat is damped by buoyancy forces before this effect on momentum transfer and the TKE balance is appreciable. He derived this expression from second-order closure equations using a coupling of the steady-state equations for TKE, density variance, and buoyancy flux. Numerous studies (e.g., Munk and Anderson 1948; Turner 1973; Launder 1975; Pacanowski and Philander 1981; Lehfeldt and Bloss 1988; Schumann and Gerz (1995); Taylor et al. 2005) have indicated that σ_*T*_ increases with the Richardson number, ${\text{Ri}} = {{{{N^2}}} \left/ {{{S^2}}} \right.} = {{{ - \frac{g}{{{\rho_0}}} \cdot \frac{{\partial \left\langle \rho \right\rangle }}{{\partial z}}}} \left/ {{{{\left| {\frac{{\partial \left\langle {{\bf U}} \right\rangle }}{{\partial z}}} \right|}^2}}} \right.}$, where *g* is the gravitational acceleration, <*ρ*> is the mean density, *ρ*_0_ is a reference density, *N* is the Brunt–Väisälä or buoyancy frequency, *S* is the vertical mean gradient of the magnitude of the mean horizontal velocity vector <**U**>, and *z* is the vertical coordinate assumed positive upwards from the bed.

Second-order closure models as presented by Ellison (1957) have been widely employed (Mellor and Yamada 1974; Launder 1975; Rohr et al. 1988; Umlauf and Burchard 2005). Ellison (1957) noted, based on the steady-state density variance balance equation alone, that buoyancy forces do not cause a transfer of heat against the gradient. However, levels 3 and 4 models of Mellor and Yamada (1974) show countergradient heat fluxes for a stratified atmospheric boundary layer, although they did not elaborate upon this finding. Since then, many studies have appeared on the countergradient buoyancy fluxes (see de Nijs and Pietrzak 2011 and references therein). The second-order closure framework shows the importance of the buoyancy correlation terms in the buoyancy flux and density variance balance equation. This complicates the analysis of the turbulent transport of mass in a density field setup by multiple active scalars, such as salt and SPM (de Nijs and Pietrzak 2011). In the case of downgradient transport this precludes the use of similar turbulent diffusivities for salinity and SPM.

Numerous geophysical studies have examined the effects of stratification on turbulence in bed boundary layer shear flows. Some have paid attention to the description of velocity shear. In atmospheric research, a log-linear profile is more appropriate for stably stratified conditions than the law of the wall (Webb 1970; Bussinger et al. 1971; Turner 1973; Dyer 1974). The log-linear profile is derived from Monin-Obukhov type dimensional analysis based on a scaling of the TKE balance (Monin and Obukhov 1954). Therefore, velocity shear near the bed depends not only on height above the bed and friction velocity, but also on the buoyancy flux. Estuarine studies by Anwar (1983) and Sanford et al. (1991) confirm this. They pointed out that the neglect of stratification by fitting a log profile can lead to overestimation of the bed shear stress and drag. When the buoyancy flux becomes negative, buoyancy becomes actively important in the production of TKE and momentum transfer. In atmospheric research, negative buoyancy fluxes are associated with unstable stratification. For this condition, the correction of the log profile results in an increase of drag instead of a decrease as for stable conditions. At large stable stratification, countergradient buoyancy fluxes are observed (de Nijs and Pietrzak 2011). These buoyancy fluxes indicate a conversion of turbulent potential energy (PE) to TKE. Therefore, they can cause an increase in TKE production and thus momentum transfer and drag. If only the damping effect is taken into account, the reduction in drag is overestimated. These consequences of a coupling with the buoyancy balance equations have not been widely considered.

**Fig. 1:**
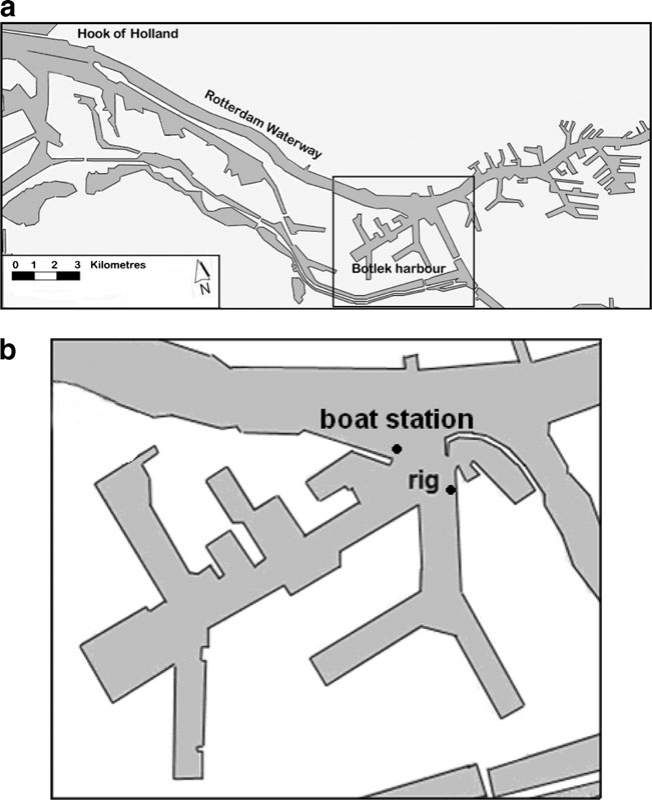
**a** The Port of Rotterdam including the Rotterdam Waterway and Botlek Harbor. **b** Location of the boat station and the rig in the turning circle of Botlek Harbor in front of a short term mooring facility for inland shipping (pontoon) to avoid interference with seagoing ship traffic. It was located at 80932 E, 433861 N about 50 m from the edge of the quay

Here, we investigate how the change of turbulence structure near the bed with stratification affects the momentum transfer, TKE production, and energy conversions. We analyze data from velocity profiles derived from acoustic Doppler current profiler (ADCP) data and from point measurements of Reynolds stresses and buoyancy fluxes collected in Botlek Harbour on April 14, 2005 and April 16, 2006. Botlek Harbour (Fig. 1) is located at the tip of the salt wedge in the Rotterdam Waterway. Section 2 presents the concept of total turbulent energy (TTE) and introduces a stratification discriminator parameter and a new expression for the σ_*T*_. We show how we can use TTE to analyze the suppression of turbulence by stratification, the consequences for the damping of vertical turbulent transports of mass and momentum and the onset of countergradient transport. In Section 3, the measuring setup and data are described. In Section 4, we characterize the variability of the turbulent flow and analyze the time evolution of the buoyancy fluxes in relation to the Reynolds shear stress and TKE. We identify different turbulence regimes and describe the energy transfers. In Section 5, we discuss our findings.

## Total turbulent energy

Here, we introduce the concept of TTE and show the importance of the conversion of energy between the vertical turbulent kinetic energy (VKE) and available turbulent potential energy (APE). We also show that the square of vertical Froude number (*F*_RV_) can be interpreted as the ratio of VKE to APE in the equation for the vertical buoyancy flux. We then derive equations for σ_*T*_ and turbulent state parameters based on VKE.

Equations for the turbulent energy, Reynolds stresses, buoyancy fluxes, and density variance can be derived from the Navier–Stokes equations for momentum, continuity, and scalar transport (Ellison 1957; Mellor and Yamada 1974; Launder 1975; Schumann 1987; Rohr et al. 1988; see Appendix). These equations are simplified here through the use of the boundary layer approximation. This approximation amounts to neglecting the advection and diffusion terms in the transport equations and is justified when the turbulent length scales are smaller than the scales associated with variations of the mean flow. Mellor and Yamada (1974) showed that the assumption of nearly homogeneous turbulence is often satisfied even in inhomogeneous boundary layers. The assumptions above result in the following set of equations

1 $$ \frac{{\partial k}}{{\partial t}} = - \left\langle {\text{uw}} \right\rangle \frac{{\partial \left\langle U \right\rangle }}{{\partial z}}\left( {1 - {R_{\text{if}}}} \right) + {\phi_k} - {\varepsilon_k} $$

2 $$ \frac{{\partial \left\langle {{u^{{^2}}}} \right\rangle }}{{\partial t}} = - 2\left\langle {\text{uw}} \right\rangle \frac{{\partial \left\langle U \right\rangle }}{{\partial z}} + {\phi_{{\left\langle {{u^2}} \right\rangle }}} - {\varepsilon_{{\left\langle {{u^2}} \right\rangle }}} $$

3 $$ \frac{{\partial \left\langle {{v^{{^2}}}} \right\rangle }}{{\partial t}} = {\phi_{{\left\langle {{v^2}} \right\rangle }}} - {\varepsilon_{{\left\langle {{v^2}} \right\rangle }}} $$

4 $$ \frac{{\partial \left\langle {{w^{{^2}}}} \right\rangle }}{{\partial t}} = - 2 \cdot {\frac{g}{\rho }_0}\left\langle {\rho w} \right\rangle + {\phi_{{\left\langle {{w^2}} \right\rangle }}} - {\varepsilon_{{\left\langle {{w^2}} \right\rangle }}} $$

5 $$ \frac{{\partial \left\langle {\text{uw}} \right\rangle }}{{\partial t}} = - \left\langle {{w^2}} \right\rangle \frac{{\partial \left\langle U \right\rangle }}{{\partial z}} - \frac{g}{{{\rho_0}}}\left\langle {\rho u} \right\rangle + {\phi_{{\left\langle {\text{uw}} \right\rangle }}} - {\varepsilon_{{\left\langle {\text{uw}} \right\rangle }}} $$

6 $$ \frac{{\partial \left\langle {\rho u} \right\rangle }}{{\partial t}} = - \left\langle {\text{uw}} \right\rangle \frac{{\partial \left\langle \rho \right\rangle }}{{\partial z}} - \left\langle {\rho w} \right\rangle \frac{{\partial \left\langle U \right\rangle }}{{\partial z}} + {\phi_{{\left\langle {\rho u} \right\rangle }}} - {\varepsilon_{{\left\langle {\rho u} \right\rangle }}} $$

7 $$ \frac{{\partial \left\langle {\rho w} \right\rangle }}{{\partial t}} = - \left\langle {{w^2}} \right\rangle \frac{{\partial \left\langle \rho \right\rangle }}{{\partial z}}\left( {1 - \frac{\text{APE}}{{\text{VKE}}}} \right) + {\phi_{{\left\langle {\rho w} \right\rangle }}} - {\varepsilon_{{\left\langle {\rho w} \right\rangle }}} $$

8 $$ \frac{{\partial \left\langle {{\rho^2}} \right\rangle }}{{\partial t}} = - 2\left\langle {\rho w} \right\rangle \frac{{\partial \left\langle \rho \right\rangle }}{{\partial z}} - {\varepsilon_{{\left\langle {{\rho^2}} \right\rangle }}} $$

where VKE is given by ½**·**<*w* ^2^> and APE = ½**·**<*ρ* ^2^>**·**(−∂<*ρ*>(∂z)^−1^)^−1^. The instantaneous value of the along-channel velocity component is the sum *U* = <*U*> + *u* of the ensemble averaged velocity <*U*> and the fluctuating velocity *u*. Similar notations are used for the cross-channel (*V*) and vertical velocity (*W*) components, salinity (*S*), and SPM (*C*). Ri_*f*_ is the flux Richardson number and represents the rate of TKE conversion by buoyancy (*B*) to the production of TKE by velocity shear (*P*)

9 $$ {\text{R}}{{\text{i}}_f} = \frac{B}{P} = \frac{{\frac{g}{{{\rho_0}}} \cdot \left\langle {\rho w} \right\rangle }}{{ - \left\langle {\text{uw}} \right\rangle {S_h}}} $$

where *S*_*h*_ is the vertical gradient of <*U*>. The variance and covariance terms are denoted as <uu> and <uw> and the TKE is indicated as *k* = ½**·**(<*u* ^2^> + <*v* ^2^> + <*w* ^2^>). ø_ij_, ø_*ρ*j_ depict redistribution of <u_i_u_j_>, <*ρ*u_j_> by pressure strain and pressure buoyancy fluctuations, respectively; *ε*_ij_, *ε*_*ρ*j_, and *ε*_(*ρ*)_^2^ are dissipation rates of <u_i_u_j_>, <*ρ*u_j_>, and <*ρ* ^2^>, respectively. The density (*ρ*) is defined as *ρ* = *ρ*_0_ + *γ*_*c*_∙*C* + *γ*_*s*_∙*S*, with γ an expression to convert salinity (*γ*_*s*_ ≈ 0.72 kg(m)^−3^(PSU)^−1^) and SPM concentration (*γ*_*c*_ = (*ρ*_*s*_ − *ρ*_*w*_)(*ρ*_*w*_)^−1^ ≈ 0.62, where *ρ*_*w*_ and *ρ*_*s*_ are the densities of water and sand particles, respectively) to density as indicated by the subscripts *s* and *c*, respectively (West and Oduyemi 1989). However, *C*<< *S* and therefore C is not separately treated in the analysis (see de Nijs and Pietrzak 2011). In Eqs. 1, 2, 3, 4, 5, 6, 7, and 8, *ρ* = *ρ*_0_ + *γ*_*s*_·*S*.

Based on energy considerations, the density variance equation (Eq. 7) can be considered a PE balance with the APE given by ½**·** *g* **·**(*ρ*)^−1^ **·** *ρ*_rms_ **·** *L*_*E*_, where *L*_*E*_ = *ρ*_rms_ **·**|(∂<*ρ*>(∂z)^−1^)^−1^| is the Ellison scale. This balance can be combined with the TKE balance (Eq. 1) to form a TTE balance *k*_tot_ = *k* + APE (Schumann 1987)

10 $$ \frac{{\partial {k_{\text{tot}}}}}{{\partial t}} = - \left\langle {\text{uw}} \right\rangle \frac{{\partial \left\langle U \right\rangle }}{{\partial z}} - {\varepsilon_k} - \frac{g}{{{\rho_0}}} \cdot \left( {\frac{{{\varepsilon_{{\left\langle {{\rho^2}} \right\rangle }}}}}{{ - \frac{{\partial \left\langle \rho \right\rangle }}{{\partial z}}}}} \right) $$

The TTE balance does not contain vertical buoyancy flux terms, signifying that the buoyancy flux converts potential energy into kinetic energy and vice versa.

### Stratification parameter

The *F*_RV_ was introduced in LES studies by Armenio and Sarkar (2002) and Taylor et al. (2005). Here, we show that $F_{\text{RV}}^2$ can be interpreted as the ratio of VKE to APE (Eq. 7)

11 $$ F_{\text{RV}}^2 = \frac{{\left\langle {{w^2}} \right\rangle }}{{{N^2} \cdot L_E^2}} = {\left( {\frac{{{L_B}}}{{{L_E}}}} \right)^2} = \frac{{\left\langle {{w^2}} \right\rangle }}{{\frac{g}{{{\rho_{{_0}}}}}{\rho_{\text{rms}}}{L_E}}} = \frac{\text{VKE}}{{\text{APE}}} $$

where *L*_*B*_ = *w*_rms_∙*N* ^−1^ is the buoyancy length scale. *L*_*E*_ is an indicator of the work required to lift a fluid parcel a distance *L*_*E*_ from its equilibrium level, and *L*_*B*_ is a measure of the vertical displacement of a fluid parcel if its VKE were converted to APE. Buoyancy becomes dynamically more important in the vertical mass transport, compared to the work by shear-produced energetic turbulence, when *L*_*B*_ becomes less than *L*_*E*_. Therefore, $F_{\text{RV}}^2$ can be considered a direct measure of the state of buoyancy on the vertical transport by energetic turbulent structures.

### Turbulent Prandtl number

The *σ*_*T*_ is defined as

12 $$ {\sigma_T} = \frac{{{\upsilon_T}}}{{{\kappa_{\rho }}}} $$

where *υ*_*T*_ is the turbulent viscosity, *κ*_*ρ*_ the turbulent diffusivity. Here, we derive an equation for *σ*_*T*_ and we show that it is not uniquely related to *R*_*i*_ and Ri_*f*_. If we aggregate the timescale effects of redistribution by pressure correlations and dissipation rates into relaxation terms (ø_ij_, ø_*ρ*j_–ε_ij_, ε_*ρ*j_ and ε_*ρ*_ = *τ* ^−1^(<u_i_u_j_>, <*ρ*u_j_>, and <*ρ* ^2^>)) and we assume a quasi-steady state (*τ* ^−1^(<u_i_u_j_>, <*ρ*u_j_>, and <*ρ* ^2^>) *τ* ^−1^>∂(<u_j_ *ρ*>, <*ρ*u_j_>, and <*ρ* ^2^>) (∂t)^−1^). This gives,

13 $$ \matrix{  {{\sigma _{T}} = \frac{{{\nu _{T}}}}{{{\kappa _{\rho }}}} = \frac{a}{{\left( {1 + c} \right)}} \cdot \left\{ {\frac{{1 - b \cdot \left( {1 - \frac{{{\text{APE}}}}{{{\text{VKE}}}}} \right)}}{{\left( {1 - \frac{{{\text{APE}}}}{{{\text{VKE}}}}} \right)}}} \right\}} \hfill \\ {{\text{with}}\;a = \frac{{{\tau _{ {\left\langle {{\text{uw}}} \right\rangle }}}}}{{{\tau _{ {\left\langle {\rho w} \right\rangle }}}}},{\text{ }}b = {\tau _{ {\left\langle {\rho w} \right\rangle }}} \cdot {\tau _{ {\left\langle {\rho u} \right\rangle }}} \cdot \left| {\frac{g}{{{\rho _{0}}}}\frac{{\partial \rho }}{{\partial z}}} \right|,{\text{ }}c = a \cdot b} \hfill \\ }<!end array> $$

An equation for the ratio APE to VKE can be constructed from Eqs. 7 and 8 by employing similar assumptions as above

14 $$ \matrix{  {\frac{{{\text{APE}}}}{{{\text{VKE}}}} = \frac{d}{{1 + d}}} \hfill \\ {{\text{with}}\;d{\text{ = 2}}{\tau _{ {\left\langle {{\rho ^{2}}} \right\rangle }}}{\tau _{ {\left\langle {\rho w} \right\rangle }}}\left| {\frac{g}{{{\rho _{0}}}}\frac{{\partial \rho }}{{\partial z}}} \right|} \hfill \\ }<!end array> $$

The coefficients *a*–*d* depend on both mean flow and turbulence properties. Therefore, these coefficients cannot be approximated in an unambiguous way. Nonetheless, we can close the timescales in Eqs. 13 and 14 with *τ* = √(*α* **·**(*S*_*h*_)^−2^) where *α* is a calibration parameter, if we assume a slower adjustment of the density variance balance with increasing stratification. It is anticipated that the effect of incomplete mixing of density fluctuations increases with *R*_*i*_ due to damping of energetic mixing which would break them down.

15 $$ {\tau_{{\left\langle {{\rho^2}} \right\rangle }}} = \tau \left( {1 + {R_i}} \right) $$

It follows from Eq. 13, regardless of the closure assumptions for the coefficients (a–c), that σ_*T*_ increases and it may become singular (σ_*T*_ → ∞) when APE∙(VKE)^−1^ approaches 1. Then it can be stated, on the basis of a quasi-steady state buoyancy balance (Eq. 7), that the buoyancy flux and Ri_*f*_ become suppressed (they approach 0). Equations 13, 14, and 15 show that *R*_*i*_ can become large provided that the σ_*T*_ increases.

### State of turbulence

Itsweire et al. (1986), Rohr et al. (1988), and Ivey and Imberger (1991) provided criteria for turbulence damping by molecular viscosity (*ν*) and a combination of stratification and molecular viscosity. In their studies, buoyancy forces to play a more prominent role than vertical velocity shear because shear is not the dominant source of turbulence. However, we must consider turbulence production by vertical shear and the vertical transport by energetic turbulent structures. Therefore, we replace the velocity scale, the centered displacement scale, and the dissipation rate of TKE (**ε**) in the expressions for the turbulent Reynolds number (Re_*T*_) and squared small-scale Froude number ((*F*_*γ*_)^2^) by the vertical root mean square velocity (*w*_rms_), a turbulence dissipation length scale *w*_rms_(|*S*_*h*_|)^−1^, and a dissipation expression based on *w*_rms_ and *S*_*h*_ (Schumann and Gerz 1995), this gives,

16 $$ {{\text Re}_T} = \frac{{{w_{\text{rms}}} \cdot {l_w}}}{\upsilon }\;{\text{with }}\;{l_{\text{w}}} = \frac{{{w_{\text{rms}}}}}{{\left| {{S_h}} \right|}} $$

17 $$ F_{\gamma }^2 = \frac{\varepsilon }{{\upsilon {N^2}}}{\text{with }}\;\varepsilon = {A_s}\frac{{{{\left( {{w_{\text{rms}}}} \right)}^3}}}{{{l_w}}} $$

**w**ith *A*_*s*_ ≈ 0.48. The criterion for the suppression of turbulence by molecular viscosity amounts to Re_*T*_ < 15 and it amounts to (*F*_*γ*_)^2^ < 15 for the suppression by a combination of molecular viscosity and stratification (Ivey and Imberger 1991). The latter criterion can be rewritten as Re_*T*_ <15**·**(*A*_*s*_)^−1^ **·** *R*_*i*_. Thus, the first and second criteria are normative when *R*_*i*_ < *A*_*s*_ and *R*_*i*_ > *A*_*s*_, respectively. The state of turbulence is examined from the Re_*T*_ vs. *R*_*i*_ diagram.

## Methods

### Location and setup

A measuring rig was deployed in the turning circle of the Botlek Harbor basin. This basin is part of the Port of Rotterdam. It is a basin adjacent to a meso-tidal estuarine channel called the Rotterdam Waterway. The rig recorded the turbulent flow structure at 0.30, 0.70, and 3.20 m above the bed (mab) using point measuring instruments from which velocity and density variances, Reynolds shear stresses, buoyancy fluxes, and production of TKE were determined; in addition, vertical profiles of shear stresses were recorded using a downward- and an upward-looking RD instruments, Inc. (RDI) 1,200 kHz Workhorse ADCPs. Six electric magnetic flow meters (EMFs), three turbidity sensors (type MEX), and three conductivity and temperature sensors were mounted on the rig and a pole attached to the rig. In addition, a boat station measured profiles of velocity and salinity for a period of 13 h in the mouth of the Botlek harbor. This data was collected with a boat-mounted ADCP 1,200 kHz type broadband and conductivity–temperature–depth sensor. Figure 1a shows the location of both the rig within Botlek Harbor and the boat station. The data were averaged with an unweighted moving average window of 10 min. A detailed description of the setup and accuracy of the data can be found in de Nijs et al. (2008, 2009) and de Nijs and Pietrzak (2011). Turbulence measurements collected from the rig on April 14, 2005 and April 16, 2006 are presented, together with the boat survey data from April 14, 2005. We anticipate that the turbulence conditions in Botlek Harbor resemble those of low tidally energetic estuarine environments, and estuarine conditions around slack water. The turbulence in estuarine environments generally occurs at higher values for shear (*U*/*H*, where *U* is a characteristic velocity scale and *H* is the water depth) and stratification (∆*ρ*/*H*, where ∆*ρ* is a characteristic density difference) than the turbulence in the ocean and shelf seas. This suggests that in estuarine environments, turbulence properties such as shear production of TKE, buoyancy fluxes and dissipation occur at a larger range of values than in ocean and shelf sea environments.

### Standard statistical analysis

The variance method is used to calculate Reynolds shear stresses from ADCP measurements, (see also Lueck and Lu 1997; Rippeth et al. 2003; Williams and Simpson 2004). Reliable estimates of Reynolds shear stresses depend on the axis of the ADCP being closely aligned with the vertical, ±4° (Simpson et al. 2005). The uncertainty associated with along beam velocity estimates are for the upward- and downward-looking ADCPs at 0.0228 and 0.006 ms^−1^, respectively (RDI Plan Software). The values of the minimal detectable stress, vertical velocity shear, and the rate of production of TKE were estimated from noise threshold level relations presented in Williams and Simpson (2004) and Simpson et al. (2005). The noise floor estimates for Reynolds shear stress, vertical velocity shear, and production of TKE for the upward- and downward-looking ADCPs amount to 2.5·10^−5^ and 1·10^−6^ m^2^s^−2^, 9.6·10^−3^ and 5.8·10^−3^ s^−1^, and 2.5·10^−7^ and 5.8·10^−9^ m^2^s^−3^, respectively. The turbulence properties acquired with the ADCPs well above the threshold levels compared favorably with those collected with the EMFs. However, at lower turbulence levels, the ADCP turbulence measurements show attenuation (biased towards 0) compared to EMF turbulence measurements at about four times the noise floor. This probably indicates that the ADCP cannot properly measure velocity fluctuations associated with turbulence structures with scales in the range of the dimensions of the bins and/or transducers. Therefore, we blank out ADCP turbulence data below four times the noise floor of the upward-looking ADCP. For the results presented here, the mean flow was homogeneous in planes along the beam spread and the tilt was not greater than 3°.

The buoyancy flux, *B* (Eq. 9) was calculated from the point measurements where *w* was measured by the EMFs and *ρ* was measured by the CT and MEX sensors. The along-channel TKE production, *P* (Eq. 9) was calculated from the vertical and along-channel velocity component measured by the horizontally-orientated EMF at 0.30 m. The cross-channel TKE production was calculated from the vertical velocity component measured by the horizontally-orientated EMF and cross-channel velocity component measured by the vertically-orientated EMF at 0.30 m. The vertical along- and cross-channel velocity shear was calculated from the velocity difference between the measuring volumes at 0.30 and 0.70 m. The cross-channel TKE production was negligible compared to the along-channel TKE production (see de Nijs 2012). Therefore, we neglect this TKE production term from the following analysis. The large-scale energetic turbulence structures contain most of the TKE. Therefore, we assume that the high-frequency losses of turbulent fluctuations as a result of sensor size and separation are not large. For a detailed discussion on the consequences of these losses for turbulence properties, see de Nijs (2012).

The *R*_*i*_ values are calculated from mean values of velocity and density. Hence, they represent average values over the temporal scales that are resolved. The sensor separation determines how well spatially values for shear and stratification are determined. To test this, we calculated *R*_*i*_ from ADCP and EMFs. The velocity distributions measured by the ADCP are more continuous than based on the EMFs at 0.30 and 0.70 m. *R*_*i*_ values based on ADCP and EMF measurements differ maximally at 30 %. Hence, the calculated values of *R*_*i*_ are somewhat sensitive to the sensor spacing. However, we believe that the calculated values are representative for those at higher spatial resolution.

### Dimensional analysis

Monin–Obukhov (1954) similarity theory is applied to the data. The dimensionless stability functions for momentum *φ*_*m*_(zL^−1^) and mass *φ*_*ρ*_(zL^−1^) are given by

18 $$ {ϕ_i} = {\left( {1 + \beta \frac{z}{L}} \right)^n}{\text{with}}\;{R_{{if}}} \approx \frac{z}{L} {\text{and}}{\left( { - < uw > \frac{{\partial \left\langle U \right\rangle }}{{\partial z}}} \right)_b} \approx \frac{{\left| {u_{*}^3} \right|}}{{\kappa z}} $$

where the subscript *i* refers to momentum or mass, *L* is the Monin–Obukhov length, and *z* is the distance above the bed. The values for *β* and n depend on the sign of zL^−1^ (Bussinger et al. 1971; Dyer 1974; Garratt 1992) and are fit to the data. The vertical profiles of velocity can be written as deviations from the logarithmic profile (Bussinger et al. 1971; Turner 1973; Dyer 1974)

19 $$ \left\langle {U(z)} \right\rangle { } = { }\frac{{{u_{*}}}}{\kappa }\left[ {\ln \left( {\frac{z}{{{z_0}}}} \right) - \lambda_m\left( {\frac{z}{L}} \right) + \lambda_m\left( {\frac{{{z_0}}}{L}} \right)} \right]{ } $$

where, λ_*m*_ is the velocity profile function and *z*_0_ is the roughness height and are used to estimate the drag coefficient, *C*_D_.

## Results

### General description of the turbulent flow characteristics

**Fig. 2:**
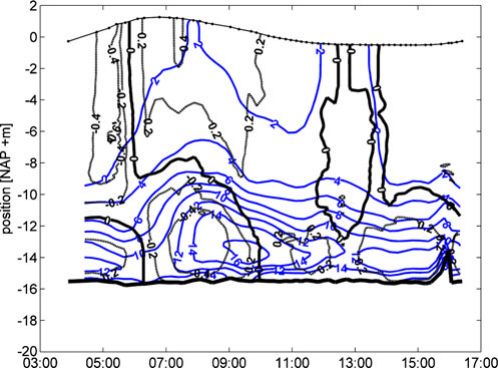
Time series of vertical profiles of salinity (psu) and velocity (ms^−1^) recorded at the boat station in the harbor mouth during the 13 h boat survey on April 14, 2005 (see de Nijs et al. 2009). The time of the survey is shown on the *x*-axis and position within the water column on the *y*-axis. The reference frame used in the Netherlands is the Normaal Amsterdamse Peil (NAP). The *blue* (*black*) contours depict salinity (primary, along channel velocity component) (positive = outflow). Note that the spike near the bed at 16:00 hours is the result of a shift in the profiling position. A spatial filter was applied to smooth the data

**Fig. 3:**
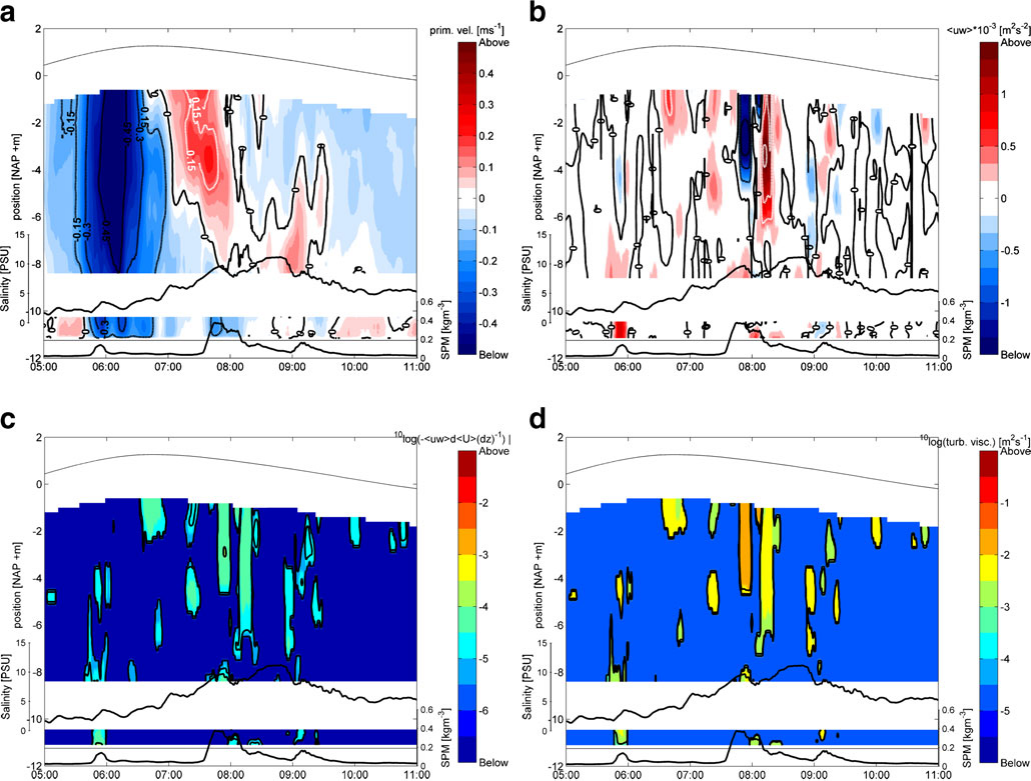
Data recorded by the ADCPs at the rig during the survey on April 14, 2005. The graphs show time series of vertical profiles of the primary (along channel) velocity component (positive = outflow; **a**), Reynolds shear stress (<uw>; **b**), the production of TKE by the Reynolds shear stress and vertical mean velocity shear (*P*; **c**), and turbulence viscosity (**d**). We blanked the data out below four times the noise floor of the upward-looking ADCP (**b**–**d**), and subsequently substituted lower threshold levels of 1e^−4^ m^2^ s^−2^, 1e^−6^ m^2^ s^−3^, and 1e^−4^ m^2^ s^−1^ for convenience of plotting. The time of the survey is shown on the abscissa and position within the water column on the ordinate. Note that **a** and **b**, **c** and **d** have different time intervals, these range from 00:00–17:00 hours and from 03:00–11:00 hours, respectively. In the lower part of graphs **a**, **b**, **c**, and **d** salinity (above) and SPM concentration (below) at 3.20 and 0.30 mab are also shown as two time series against a secondary *y*-axis on the left and right, respectively. The water level and harbor bed are shown by the thin black lines. The band within −10 and −8 m (**a**–**d**) is due to the blanking distances of the upward and downward-looking ADCPs. These distances amount to 1.8 and 0.50 m, respectively. Thus, a part of the water column is not measured

Under typical conditions (de Nijs et al. 2008, 2009), a lock-exchange mechanism controls the exchange of saline and turbid water between the Botlek Harbor and the Rotterdam Waterway. During the flood, the salt wedge and estuarine turbidity maximum (ETM) appear in front of the mouth of the Botlek Harbor (de Nijs et al. 2010, 2011; Fig. 2). Then, a lock-exchange mechanism acts such that denser saline waters, containing SPM (not shown), intrude into the harbor in the lower layer and fresher waters outflow into the Rotterdam Waterway in the surface layer (06:00–10:00 hours). During the ebb, the salt wedge and ETM are advected down estuary of the Botlek Harbor. Hence, the water at the mouth becomes fresher and the lock-exchange reverses direction (13:00–17:00 hours). TKE and vertical turbulent transport of momentum increase during the rising tide and the intrusion of the density current (see Fig. 3a in de Nijs and Pietrzak 2011). The salinity structure shows a weakly stratified to well-mixed upper layer, separated by an interface at approximately −8.0 to −10.0 m Normaal Amsterdamse Peil (NAP) from a lower layer of stratified saltier water. Note the almost linear increase in salinity towards the bed in the lower layer. The vertical salinity distribution is similar to that observed at a station close to the rig, although here the interface (referred to hereafter as the pycnocline) is at approximately −8.0 m NAP, see also Fig. 6b in de Nijs et al. (2009).

### Vertical distribution of turbulence quantities

The time development of the mean and turbulent flow at the rig are presented in Fig. 3. Figure 3a shows that from 05:00–05:40 hours an exchange flow is present with outflow in the lower layer. However, by ~05:40 inflow (tidal filling) over the entire water column is evident, this persists until ~07:00 hours when an exchange flow develops, but now with inflow in the lower layer which lasts until ~09:00 hours. Thereafter, the exchange flow reverses direction, but the velocities are now much smaller. Turbulence levels are generally low throughout the water column but local areas of relatively high Reynolds stresses are observed (Fig. 3b), which generally correlate with areas of high production of TKE (Fig. 3c). Note the removal of data well above the noise floor levels explains why ADCP shear production remains positive. Turbulent viscosities (Fig. 3d) are generally low (below the measuring threshold) throughout most of the water column while during tidal filling values range from 5·10^−4^ to 4·10^−3^ m^2^ s^−1^ and during the intrusion of the density current turbulent viscosity values vary between about 0.5·10^−4^ to 5·10^−3^ m^2^ s^−1^.

The dominant source of turbulence is production by vertical shear both near the bed (05:40–06:00 and 08:00–08:30 hours), near the pycnocline (05:40–06:00, ~06:30, 07:30–08:00, ~08:45, and ~09:05 hours) and in the upper water column. However, the patches of high Reynolds stresses in the uppermost 2 m of the water column could be influenced by wakes from ship’s propellers. Near the pycnocline, the mean stratification was unstable at ~06:00 and ~08:00 hours (not shown). This indicates the presence of overturning instabilities (see de Nijs and Pietrzak 2011).

During tidal filling at ~06:00 hours, distinct Reynolds shear stresses and turbulence viscosities are confined to the lower part of the water column between the bed and ~NAP −8 m. During tidal emptying at ~08:00 hours, shear due to the exchange flow leads to distinct Reynolds stresses and turbulence viscosities between the near surface and NAP −5 m and between the bed and NAP −8 m (Fig. 3b,d). These areas are separated by an area with significantly less turbulence at intermediate depths, indicating that the stratification act as turbulent energy barriers for bed-generated turbulence. The near-bed measurements show stable mean stratification throughout the survey period, with N^2^ ranging from 0.02 to 0.05 s^−2^.

### Buoyancy fluxes

**Fig. 4:**
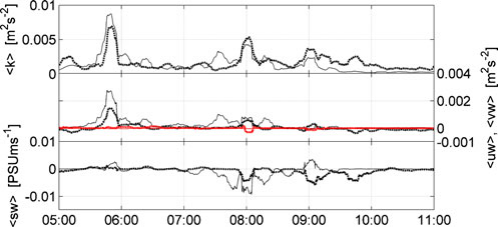
Data recorded by point measuring instruments (EMFs, conductivity, temperature and MEX sensors) at the rig during the survey on April 14, 2005. The graph shows TKE, vertical turbulent transport of momentum (<uw> (*black lines*), <vw> (*red lines*)), vertical turbulent transport of salinity (<sw>), respectively. Time (hours) is shown on the *x*-axis. Data at 0.30 mab (*dashed thick line*) and 3.20 mab (*thin line*) are shown

It is only near the bed that we have buoyancy flux data. The corresponding time development of the SPM fluxes measured at 0.3 and 3.2 m above the bed are shown in de Nijs and Pietrzak (2011). Here we reproduce the TKE, Reynolds shear stresses (<uw>), and salinity fluxes and include <vw> to show that the cross-channel contribution is negligible (Fig. 4). TKE remains active (first panel) and Reynolds shear stresses (second panel) remain downgradient throughout the survey period. The sign changes of <sw> indicate that buoyancy fluxes can act as a sink or source of TKE.

**Fig. 5:**
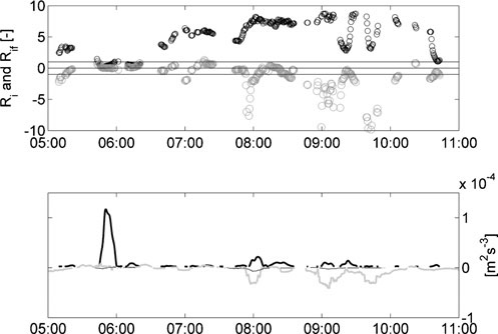
Turbulence measurements recorded by point measuring instruments (EMFs, conductivity, temperature and MEX sensors) at 0.30 mab at the rig on April 14 2005 (about 22 h of turbulence measurements). The *upper graph* shows Ri_*f*_ (*gray*), *R*_*i*_ (*black*). The *thin lines* denote either *R*_*i*_ or Ri_*f*_ = 1 and *R*_*i*_ or Ri_*f*_ = −1. The lower graph shows the buoyancy flux caused by SPM (*thin black line*), buoyancy flux caused by salinity (*thick gray line*) and production of TKE by vertical shear (*thick black line*)

The negative buoyancy fluxes at the pycnocline at 06:30 and 07:30 hours are downgradient associated with unstable mean stratification, while those at other times and near the bed are countergradient. The ratio of APE to VKE determines the transition between down- and countergradient buoyancy fluxes, see Fig. 5 in de Nijs and Pietrzak (2011). The negative salinity fluxes signify an active contribution by buoyancy to the turbulent transfer of energy and mass. The observations show a maximal value of the buoyancy flux due to salinity *B* ≈ −3.6·10^−5^ m^2^ s^−3^. It is about 25 % of the maximal value found for TKE production by shear *P* ≈ 1.2·10^−4^ m^2^ s^−3^. However, these values do not coincide. Thus, at some instances, the turbulence field is actively controlled by buoyancy. Negative buoyancy flux values caused by salinity are a factor 10 to 100 larger than those caused by SPM (de Nijs and Pietrzak 2011). Hence, buoyancy fluxes due to SPM do not significantly affect the TKE budget compared to buoyancy fluxes due to salinity. Hereafter, we refer to buoyancy fluxes as due to salinity. At the pycnocline ~3.20 mab from 05:40–06:40 and ~07:40 hours larger values for <uw> (Figs. 3b and 4) are found than at 0.30 mab. Figure 3c shows that at the pycnocline the production of TKE by high vertical shear is significant, but the TKE locally generated by negative buoyancy fluxes needs to be considered as well (Fig. 4, third panel).

### Characterization of turbulent conditions

Here, we characterize the turbulence conditions by examining the stability of the flow using *R*_*i*_ and Ri_*f*_ numbers, and the relative competition between production of TKE due to *P* and the source and sink function of the *B*. The Ri_*f*_ and *R*_*i*_ numbers at 0.30 mab are shown in Fig. 5 (upper graph). The positive values for Ri_*f*_ range from 0.08 (around 05:50 hours) to 1.2 (around 05:55 and 07:30 hours) and values for *R*_*i*_ range from 0.15 (around 05:50 hours) to 8 (around 09:00 hours). Figure 5 shows that almost throughout the entire measuring period, values of *R*_*i*_ generally exceed *R*_*i*;crit_ (0.25–1) at which turbulence is damped (Miles 1961; Gerz and Schumann 1996). Between the time period 05:40–06:30 hours, values of *R*_*i*_ are below *R*_*i*;crit_. Then Ri_*f*_ values are generally positive and above Ri_*f*;crit_ (≈0.15; Turner 1973; Osborn 1980; Nieuwstadt 1998). During much of the time before and after the period of weaker stratification, values of Ri_*f*_ are generally countergradient. However, some positive Ri_*f*_ values are observed (around 06:50, 07:15, 08:15, and 10:15 hours). Hence, the turbulent flow can be characterized as strongly stable where the turbulent structure and hence the turbulent mass and momentum transport are strongly affected by buoyancy forces. Note that most of the large positive Ri_*f*_ numbers correlate with relatively weak production of TKE by shear (around 05:40, 06:20, 07:15 hours). For quasistationary conditions, the large Ri_*f*_ numbers of about and above one indicate low dissipation rates (Eq. 1, ε ≈ P(1 − Ri_*f*_)) and significant effects of buoyancy on the TKE budget. However, nonstationary effects associated with the turbulence field should also be taken into account.

Periods when Ri_*f*_ < 0 (between 05:00–05:30, 06:40, 07:00–07:10, 07:50–08:10, and 08:20–11:00 hours) indicate conversions of APE by countergradient buoyancy fluxes into VKE. Periods when Ri_*f*_ > 0 (between 05:40–06:30 and around 06:50, 07:15, 08:15, and 10:15 hours) depict conversions of VKE by positive buoyancy fluxes resulting from transport by energetic turbulent structures into APE (de Nijs and Pietrzak 2011). Furthermore, when |Ri_*f*_| < 1 production of TKE by shear dominates the TKE balance over production by buoyancy (APE conversion into VKE) or destruction by buoyancy (VKE conversion into APE), while for conditions when |Ri_*f*_| > 1 conversion by buoyancy dominates the TKE balance over production by shear. It can be seen from upper graph of Fig. 5 that the latter condition mostly occurs. Periods when Ri_*f*_ < −1 indicate that buoyancy is dynamically important in the transport of mass and momentum.

### Scaling analyses

**Fig. 6:**
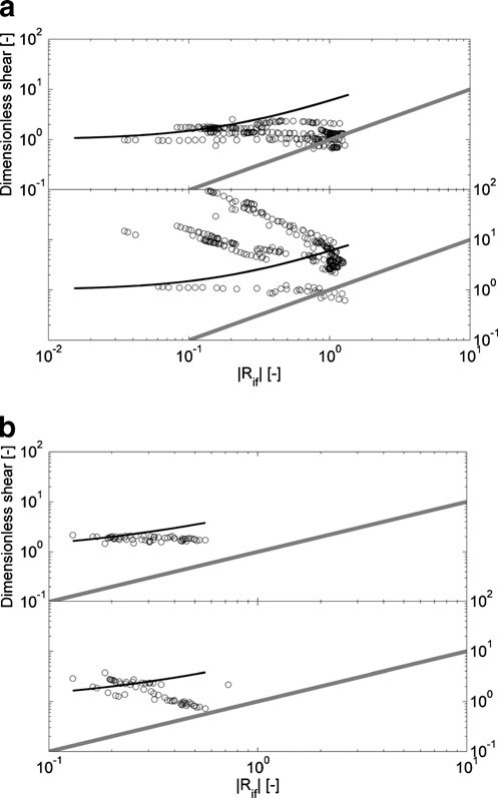
The graphs show dimensionless velocity (*upper panel*) and density gradients (*lower panel*) as a function of the absolute value of Ri_*f*_ for Ri_*f*_ > 0 calculated from data at 0.30 mab recorded by point measuring instruments (EMFs, conductivity, temperature and MEX sensors) at the rig on April 14, 2005 (**a**) and on April 16, 2006 (**b**). The *black lines* denote stability functions (*φ*_*m*_(zL^−1^), *φ*_*ρ*_(zL^−1^)) and the slope of the *gray lines* denote the power of the stability functions

**Fig. 7:**
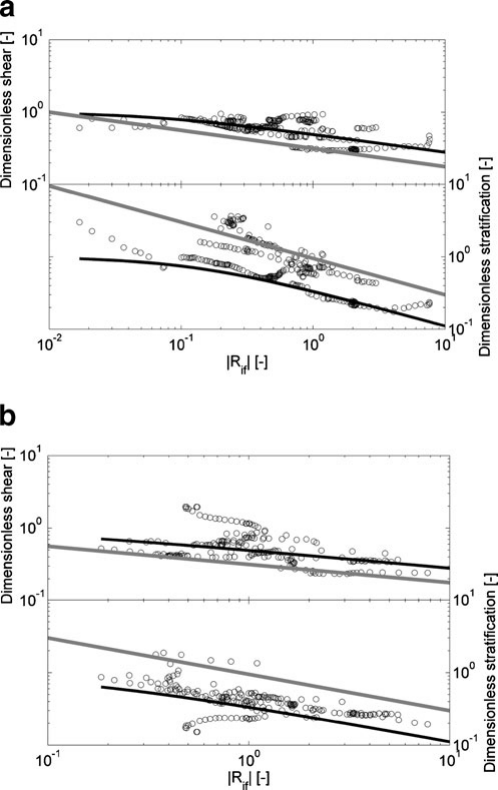
The graphs show dimensionless velocity (*upper panel*) and density gradients (*lower panel*) as a function the absolute value of Ri_*f*_ for Ri_*f*_ < 0 calculated from data at 0.30 mab recorded by point measuring instruments (EMFs, conductivity, temperature and MEX sensors) at the rig on April 14, 2005 (**a**) and on April 16, 2006 (**b**). The *black lines* denote stability functions (*φ*_*m*_(zL^−1^), *φ*_*ρ*_(zL^−1^)) and the slope of the *gray lines* denote the power of the stability functions

**Fig. 8:**
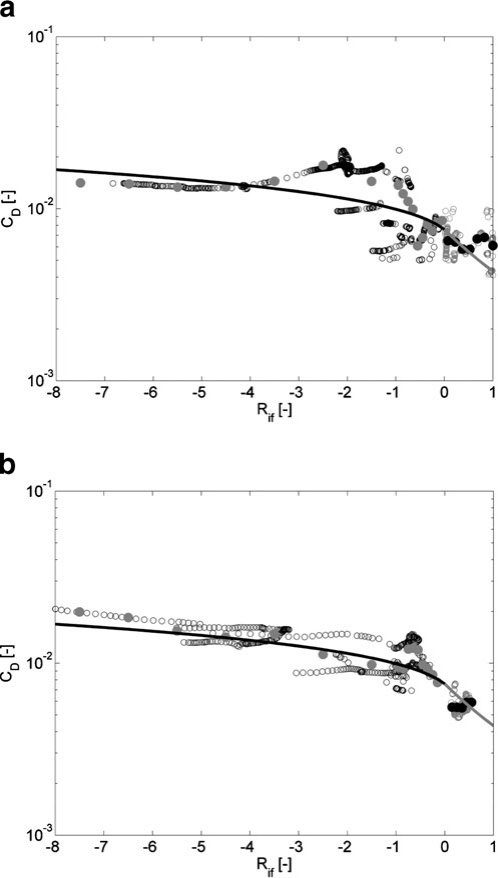
The graphs show drag coefficient (*C*_*D*_) as a function of Ri_*f*_ (≈zL^−1^) calculated from data at 0.30 mab recorded by point measuring instruments (EMFs, conductivity, temperature and MEX sensors) at the rig on April 14, 2005 (**a**) and on April 16, 2006 (**b**). The drag coefficient is defined as the ratio of the near-bed Reynolds shear stress (<uw>_*b*_) to the square of the along-channel near-bed velocity. The *black* (*gray*) symbols denote *C*_*D*_ values for zL^−1^ < 0 or Ri_*f*_ < 0 (zL^−1^ > 0 or Ri_*f*_ > 0). The *black (gray*) *lines* denote drag coefficient values calculated with profile functions for zL^−1^ < 0 or Ri_*f*_ < 0 (zL^−1^ > 0 or Ri_*f*_ > 0). The *thick black* (*gray*) *dots* are binned values of C_D_ for zL^−1^ < 0 or Ri_*f*_ < 0 (zL^−1^ > 0 or Ri_*f*_ > 0)

The Monin–Obhukov dimensional analysis (Figs. 6, 7, and 8) is carried out to determine the active role of buoyancy in the transport of momentum. Figures 6 and 7 show values of dimensionless vertical velocity shear and stratification. Their trends follow the dimensionless stability functions *φ*_*m*_(zL^−1^) and *φ*_*ρ*_(zL^−1^) with *β* = 5 and *n* = 1, with *β* = 5 and *n* = 1 for Ri_*f*_ > 0 and with *β* = −16 and *n* = −0.25, with *β* = −8 and *n* = 0.25 for Ri_*f*_ < 0. Hence, the measurements demonstrate that vertical velocity shear and stratification depend on height above bed and shear velocity, as well as on the buoyancy flux. However, the data, particularly those related to the density field do not appear to entirely follow Monin–Obhukhov scaling.

Figure 8 shows that the trend of calculated dimensionless drag coefficients (*φ*_*m*_(zL^−1^) and *z*_0_ = 0.003 m) are in reasonable agreement with drag coefficients derived from measurements. However, the quantitative agreement is less satisfactory. Nonetheless, the measurements demonstrate that the drag coefficient is lower and higher than for neutral conditions when Ri_*f*_ > 0 and Ri_*f*_ < 0, respectively. When zL^−1^ > 0 *C*_*D*_, decreases with increasing zL^−1^. Then, a log-profile fit would overestimate the value of *C*_*D*_ and <uw>_*b*_. For negative zL^−1^ < 0 *C*_*D*_, increases with increasing |zL^−1^|. Then, a log-profile fit would underestimate the value of *C*_*D*_ and <uw>_*b*_. The latter behavior of *C*_*D*_ with Ri_*f*_ (or zL^−1^) is associated with unstable conditions (*R*_*i*_ < 0) in most atmospheric studies. However, *R*_*i*_ was always positive at 0.30 mab. Thus, the behavior of C_D_ with Ri_*f*_ (or zL^−1^) cannot be unambiguously parameterized using *R*_*i*_.

**Fig. 9:**
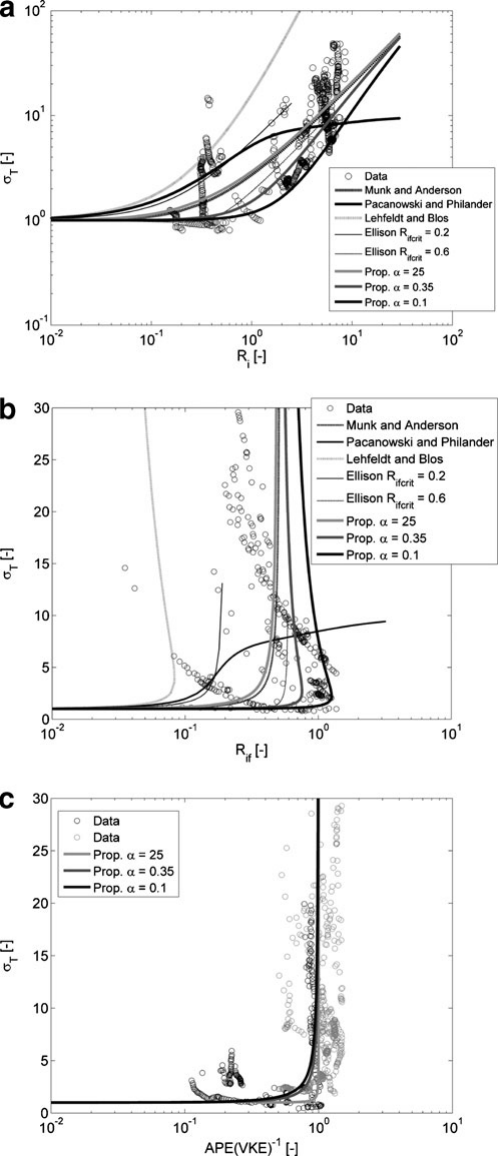
Graph **a** shows σ_*T*_ vs. *R*_*i*_, graph **b** shows σ_*T*_ vs. Ri_*f*_, and graph **c** shows σ_*T*_ vs. APE(VKE)^−1^. These quantities were computed from turbulence measurements recorded by point measuring instruments (EMFs, conductivity, temperature and MEX sensors) at the rig at April 14, 2005 (about 22 h of turbulence measurements). It is noted that the calculated σ_*T*_ vs. APE(VKE)^−1^, which had disputable statistical significance, were left out of the analysis at first. This resulted in much loss of data. For the purpose of the analysis, these data have been included in the graph indicated by color *gray*, while statistical significant data are depicted by the color *black*

### Turbulent Prandtl values

The time variations and values of Ri_*f*_ and *R*_*i*_ are similar between 05:45 and 06:15 hours, while between 06:30–07:30 and 08:00–08:30 hours *R*_*i*_ > Ri_*f*_. This implies that σ_*T*_ is larger than 1. Furthermore, at other times, positive values of *R*_*i*_ correlate with negative values of Ri_*f*_. Then, the gradient type transport hypothesis has broken down. These trends of Ri_*f*_ with *R*_*i*_ indicate that a consistent relationship between Ri_*f*_ and *R*_*i*_ cannot be observed from Fig. 5. Hence, they indicate that a unique value for σ_*T*_ does not exist. Here, we explore the behavior of σ_*T*_ with *R*_*i*_, Ri_*f*_ and *F*_RV_ for downgradient buoyancy fluxes. On the basis of Eq. 12, it can be inferred that it can increase with *R*_*i*_ or with the decrease of Ri_*f*_. Indeed, this trend can be observed in Fig. 9a, b. It shows that σ_*T*_ increases with increasing *R*_*i*_ and decreasing Ri_*f*_, respectively. However, the data trend in the figures suggests that σ_*T*_ is not uniquely related to either *R*_*i*_ or Ri_*f*_.

**Fig. 10:**
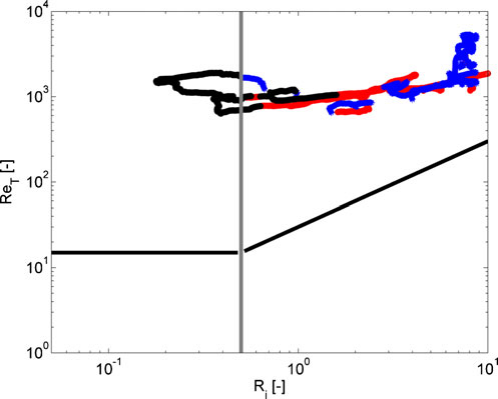
Turbulent Reynolds number vs. *R*_*i*_. The *thick black lines* denote the boundaries beyond which turbulence is damped due to molecular viscosity and by a combination of molecular viscosity and stratification. *Blue* data where *B* < 0, the *black* data where *B* > 0, *red* data of <*w* ^2^> below detection limits. These quantities were computed from turbulence measurements recorded by point measuring instruments (EMFs, conductivity, temperature and MEX sensors) at the anchored station on April 14, 2005 (about 22 h of turbulence measurements)

Notably, the Munk and Anderson (1948) relationship and the proposed relationships (Eqs. 13, 14, and 15) are in agreement with the gross trend of the data. The relationship by Ellison (1957) with Ri_*f*;crit_ = 0.6 describes this trend reasonably well, but not for smaller values of Ri_*f*;crit_. The relationships of Pacanowski and Philander (1981) and Lehfeldt and Bloss (1988) do not fit the data. A Prandtl number based on Monin–Obukhov similarity (σ_*T*_ = (*φ*_*ρ*_)(*φ*_*m*_)^−1^, not shown) does not fit the trend of the data either (Fig. 9b). Figure 9c shows σ_*T*_ versus the ratio APE to VKE. On the basis of Eq. 13, it can be inferred that σ_*T*_ increases when this ratio increases to about 1. Moreover, σ_*T*_ should strongly increase when the ratio of APE to VKE approaches 1. The data and proposed relationships plotted in Fig. 9c support this trend. The above indicates that the behavior of σ_*T*_ is in agreement with a coupling of the Reynolds stress, covariance, and variance balance equations. Thus, knowledge of the processes involved in the damping of turbulent momentum transport is not sufficient to deduce the processes involved in the damping of turbulent transport of buoyancy.

### Conceptual picture of the energy transfer

**Fig. 11:**
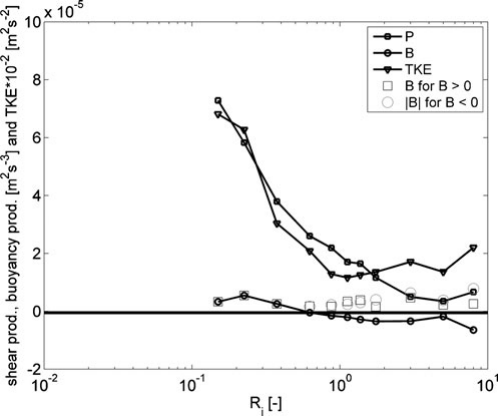
Binned values of the TKE production (P, *line with squares*) and turbulent energy conversion by buoyancy (*line with circles*) and TKE (*triangle*) vs. *R*_*i*_. The *black squares* denote values of *B* > 0 and the *gray* circles denote absolute values of *B* < 0. These quantities were computed from turbulence measurements recorded by point measuring instruments (EMFs, conductivity, temperature and MEX sensors) at the rig on April 14, 2005 (about 22 h of turbulence measurements)

Here, the turbulence regime and the consequences for energy transfer are discussed for the 2005 measurements using Re_*T*_, Ri_*f*_, *R*_*i*_, and (*F*_RV_)^−2^. The turbulence state diagram (Fig. 10) shows that with increasing *R*_*i*_, the values of Re_*T*_ remain above the viscous limit. Thus, buoyancy forces determine the decay of turbulence. Indeed, values of <*w* ^2^> below the detection limit and countergradient buoyancy fluxes for values of *R*_*i*_ > 1 imply strong effects of buoyancy forces on the turbulence structure. However, turbulent Prandtl numbers (Fig. 9a) and TKE (Fig. 11) far beyond the critical thresholds for *R*_*i*_ signify that turbulence remains active. A criterion for the damping of TKE can be derived from the energy equation (Eq. 1) by neglecting dissipation and pressure strain transfer. Then, TKE would decay if *R*_*if*_ ≥ 1 and if the buoyancy flux remains positive. This criterion is an upper limit estimate of Ri_*f*;crit_ for the TKE balance. This can be qualitatively explained by considering that TKE is put initially into <*u* ^2^> by vertical shear (Eq. 2) and subsequently it is redistributed through pressure–strain correlations (Launder 1975) to the <*v* ^2^> and <*w* ^2^> balances (Eqs. 3 and 4). The TKE is dissipated by molecular viscosity at all three components and buoyancy only affects <*w* ^2^> directly. This suggests that the Ri_*f*;crit_ for the <*w* ^2^> balance (Eq. 3) is lower than for the *k* balance (Eq. 1). Furthermore, it indicates that only a part of the TKE budget is indirectly transferred to the <*ρ* ^2^> balance through positive buoyancy fluxes. The above analysis indicates that the turbulent transfer of mass is suppressed by buoyancy forces before buoyancy fluxes become countergradient, and before the TKE transfer and TKE balance are suppressed. Thus, it is unlikely that all turbulence quantities (Eqs. 1, 2, 3, 4, 5, 6, 7, and 8) are damped simultaneously or that a single critical value of Ri_*f*_ and *R*_*i*_ exists to characterize the turbulent flow field.

**Fig. 12:**
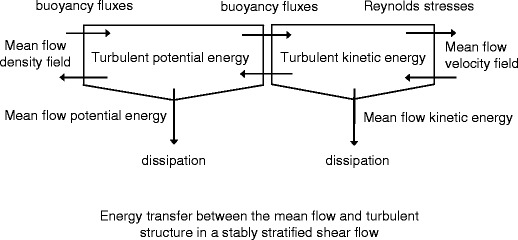
Schematic of the energy transfer between the mean flow and turbulence field in buoyancy affected and controlled flows. When the flow is affected by buoyancy and stratification, ensemble mean flow energy is transferred by Reynolds shear stresses and shear to the TKE balance. This TKE drives energetic turbulent structures that mix scalar quantities through positive buoyancy fluxes. These buoyancy fluxes increase APE and potential energy of the mean flow at the expense of VKE. Part of the APE is stored, while another part is diffused and dissipated. The ratio APE to VKE increases with stratification (de Nijs and Pietrzak 2011). However, when buoyancy starts to control the turbulent flow at strong stratification, the ratio between APE and VKE in the balance equation of the buoyancy flux (Eq. 7) develops such that the vertical turbulent buoyancy flux becomes countergradient. Then, the countergradient buoyancy fluxes transfer APE to VKE. Therefore, they act to restore some kind of equilibrium between APE and VKE. Then, the TKE and Reynolds stresses increase, even though density gradients sharpen and mean flow potential energy is converted

Next, we examine the gross effects of stratification on the turbulence structure from binned quantities of *P* and *B* vs. *R*_*i*_ (Fig. 11). This plot shows that with increasing stratification, the TKE and TKE production are suppressed, but they do not cease. The conversion of TKE by positive buoyancy fluxes into turbulent potential energy occurs at all *R*_*i*_. In the mean, however, at *R*_*i*_ < 0.6, the conversion is predominantly from the TKE balance into the turbulent potential energy balance, while at *R*_*i*_ > 0.6, the conversion is predominantly from the potential energy balance into the TKE balance. At *R*_*i*_ > 3, the conversion by buoyancy is of the same order of magnitude as the TKE production by shear.

Here, we discuss the conceptual picture that emerges about the energy transfer in flows determined by buoyancy forces shown in Fig. 12. The mathematical background is presented in Section 2 and Appendix. Around 06:00 hours (Figs. 4 and 5), the turbulence structure can be characterized as affected by buoyancy. This means active mixing by turbulence generated at the bed as a result of velocity shear caused by the rising tide (*R*_*i*_ < *R*_*i*;crit_, Ri_*f*_ ≤ Ri_*f*;crit_, (*F*_RV_)^−2^ < 1, VKE > APE, *B* > 0, |*B*| < |*P*|). However, vertical turbulent transports are affected by stable stratification. In this regime, the energy extracted from the ensemble mean flow by *P* is put directly into the balance of <*u* ^2^>. Subsequently, it is redistributed over the balances of <*v* ^2^> and VKE through pressure–strain transfer. *A*_*s*_ a result of stratification, positive *B* removes energy from the balance of VKE. This energy is transferred by positive *B* into the balance of APE.

With increasing *R*_*i*_, VKE starts to decay in the case of the removal of energy from the VKE balance by positive *B* exceeds the input of TKE by pressure–strain transfer. The positive *B* cause APE (Eq. 8) to increase. At some point, the balance between VKE and APE in Eq. 7 may shift causing countergradient *B*. Then, the turbulence structure can be characterized as buoyancy controlled (*R*_*i*_ > *R*_*i*;crit_, Ri_*f*_ < 0, (*F*_RV_)^−2^ > 1, APE > VKE, *B* < 0, |*B*| > |P|). This occurs around 05:50 and 07:40 hours. In this regime, countergradient *B* acts as a source of TKE in the balance equation of VKE. Hence, energy from the APE balance is directly transferred into the balance VKE by countergradient *B*. Subsequently, some of this energy is removed from the balance of VKE through pressure strain transfer into the balances of <*u* ^2^> and <*v* ^2^>.

## Discussion

In this paper, we present a further analysis of observations of countergradient buoyancy fluxes presented in de Nijs and Pietrzak (2011) and show that the characteristics of turbulence can be explained by the concept of total turbulent energy. This concept allows us to distinguish between vertical and horizontal TKE and PE, and we associate the vertical component with vertical transport. The near-bed turbulence is determined by alternate periods where the buoyancy flux acts either as a source or a sink of VKE. In the case of a source it converts APE to VKE, and in the case of a sink it converts it to APE. Measurements show that saltwater can extend into the upper part of the water column (Fig. 2, ~07:00 and ~13:00 h at ~2 to 3 m below the surface, and see de Nijs et al. 2009). Hence, conversions of turbulent kinetic and potential energy are likely to contribute to the turbulence structure higher in the water column. Note that while TKE and Reynolds shear stresses indicate localized energetic turbulence and downgradient transfer of momentum, the vertical countergradient buoyancy fluxes due to salinity cause restratification. Countergradient buoyancy fluxes due to SPM, although lower in magnitude (not shown), also cause restratification and enhanced settling (de Nijs and Pietrzak 2011). The stratification near the bed and pycnocline confines bed-generated turbulence to the lower part of the water column indicating a reduced SPM-carrying capacity of the flow and subsequent settling of SPM above it. In the pycnocline and at localized areas higher in the water column, high shear stress events occur. Some of these events correlate with time mean unstable stratification indicating the presence and remnants of overturning instabilities (see de Nijs and Pietrzak 2011) possibly due to Kelvin–Helmholtz instability (e.g., Geyer and Smith 1987; Thorpe 1998). Near the bed, the mean stratification remained stable. Here, vertical shear production is the main source of local turbulence around 05:50 hours (*R*_*i*_ < *R*_*i*;crit_) while buoyancy production dominates around 07:40 hours (*R*_*i*_ > *R*_*i*;crit_).

Dimensional analyses Monin–Obukhov (1954) corroborates both the mean passive (suppression) and the dynamical active (production) role of buoyancy in the vertical turbulent transport of mass, production of TKE and consequently momentum transfer near the bed. The data are not fully Monin–Obukhov self similar, particularly the density data. This is consistent with deductions that the turbulence field is not in equilibrium or steady state. Values of Ri_*f*_ around 1, and the occurrence of turbulence for values of *R*_*i*_ beyond critical thresholds of 0.25 and 1 (Miles 1961; Gerz and Schumann 1996) indicate that turbulence decays. Moreover, the countergradient buoyancy fluxes act to restore some kind of equilibrium between APE and VKE.

The physical explanation for the countergradient buoyancy fluxes near the bed is an increase in the importance of the asymmetry in transport by energetic shear-driven turbulent structures as a result of buoyancy forces and an increase of the contribution of incompletely mixed buoyant parcels of fluid to the mean turbulent transport of buoyancy with increasing stratification. At large *R*_*i*_, the contribution of the shear-driven energetic motions to transport will reduce compared to contributions by buoyantly moving parcels. This results in mean countergradient turbulent buoyancy fluxes persisting longer than half the buoyancy period. This distinguishes them from those created at the buoyancy frequency by internal waves. In stratified flows, internal waves and turbulence coexist (e.g., Jacobitz et al. 2005). Therefore, convective motions can also result from unstable or breaking internal waves (e.g., Geyer and Smith 1987; Thorpe 1998). We believe that the countergradient buoyancy fluxes at the pycnocline may be viewed as remnants of Kelvin–Helmholtz instabilities.

Our view is that these buoyant parcels represent a memory effect because they retained some information of their origin from which they were transported by energetic turbulence. This memory lasts longer for density fluctuations than for velocity fluctuations. Momentum can be transferred relatively quickly with the surrounding fluid through pressure fluctuation forces (collisions). In contrast, active scalar (salt, SPM) transfer occurs more slowly because it requires mixing of fluid parcels with their surroundings in order to achieve irreversible exchange. Hence, the age or lifetime of these parcels increases relative to that of energetic turbulence structures with increasing stratification. This highlights the importance of (1) a slower relaxation of the density variance balance with increasing stratification (Eq. 15) and (2) contributions by the local rate of change terms in the variance and covariance balances (Eqs. 7 and 8). This in turn would indicate some finite adjustment time of the turbulence structure to the variations of the mean velocity and density gradients.

TKE and values of the Prandtl number and beyond the critical thresholds for *R*_*i*_ of 0.25–1 (Miles 1961; Gerz and Schumann 1996) show the importance of energetic turbulent transports at large stratification. The critical Richardson number of 0.25 by Miles (1961) is a linear stability threshold for a steady two-dimensional stably-stratified horizontal shear flow, while turbulent flows are 3D nonlinear stochastic phenomena characterized by 3D vorticity fluctuations. Therefore, the application of the linear threshold by Miles (1961) is not straight forward. Gerz and Schumann (1996) applied *R*_*i*_ thresholds based on average values to characterize turbulence conditions from unsteady nonlinear analyses (LES) and measurements. Thus, intermittent processes are averaged out. This definition is consistent with a number of studies (e.g., Komori et al. 1983; Rohr et al. 1988; West and Shiono 1988; Taylor et al. 2005). We consider the thresholds by Gerz and Schumann (1996) more appropriate to characterize our turbulence conditions. In their investigations, *R*_*i*_ ranges from 0 to 1 and also do not show complete suppression of TKE. Neither a single critical value beyond which turbulence is completely suppressed (see also Section 4.7) nor a single discriminator parameter may exist to characterize this (Section 2). Hence, energetic turbulence may occur for even larger *R*_*i*_. This is consistent with LES results (Taylor et al. 2005) and it is in agreement with the position of the data in the Re_*T*_ vs. *R*_*i*_ plot. Figure 11 shows that the flow structure remains turbulent. The relation for σ_*T*_ supports the view that the buoyancy flux balance is already affected by buoyancy forces before momentum transfer (e.g., Munk and Anderson 1948; Turner 1973; Pacanowski and Philander 1981) and the TKE budget (Ellison 1957) are affected. This mathematical behavior is consistent with the conceptual view presented in de Nijs and Pietrzak (2011) about the motions that determine the instantaneous transports of momentum, salinity, and SPM. Buoyancy forces create countergradient transport of buoyancy before the damping of vertical turbulent transport of momentum is appreciable.

The countergradient buoyancy fluxes and turbulent Prandtl numbers above 1 with increasing *R*_*i*_ are consistent with TTE. The closure relations in this set of balance equations should reflect the dynamics of the intermittent motions with stratification. This determines terms such as pressure–strain transfer, diffusion, and dissipation, which are different for velocity, scalar fluctuations, and over the range of turbulent scales (Gerz and Schumann 1996; de Nijs and Pietrzak 2011). The TTE model (Section 2) can serve as an alternative turbulence model (subgrid model) in LES and RANS models to account for reversible contributions to mixing. However, these turbulence models would require different closure assumptions in the balance equations because the motions that contribute to countergradient transport are dynamically different at the large and small scales. Two-equation turbulence models in combination with turbulent Prandtl closures are commonly used to model vertical turbulent transports. On the basis of Eqs. 1, 2, 3, 4, 5, 6, 7, and 8, it could be argued that the *k* balance should reflect the dynamics of the balance of *w* ^2^. Furthermore, the two-equation models cannot reproduce the energy conversion between the PE and TKE balances. Our measurements further show for positive Ri_*f*_ that the turbulent Prandtl number increases with *R*_*i*_. Some models do not account for this behavior. Moreover, the modeling of mixing of two potentially active scalars using the turbulent Prandtl number analogy may be somewhat ambiguous. This holds for second-order closure modeling as well, since closures need to be specified for the buoyancy covariance terms which express the interactions between the active scalars (see de Nijs and Pietrzak 2011). Further note that second-order models and two-equation turbulence models such as the *k*–ε model employ boundary conditions based on the assumption of equilibrium between production and dissipation. However, additional terms such as buoyancy terms determine their balances. Moreover, the measurements suggest that Eqs. 1, 2, 3, 4, 5, 6, 7, and 8 cannot generally be considered in a steady state.

## Appendix

### Energy transfer between the mean flow and turbulent structure in a stably stratified shear flow

The turbulent kinetic and potential energy transfer between the mean and turbulent flow can be investigated with the Navier–Stokes (NS) and Reynolds-averaged Navier–Stokes (RANS) equations for mass and momentum conservation. In these equations, the velocity and density field are written as the sum of an ensemble average (mean flow) plus a deviation (turbulent fluctuation). The mean flow equations for kinetic and available potential energy (MKE and MPE) can be obtained by multiplying the RANS momentum and mass balances with <*U*_*i*_> and <*ρ*>, respectively. The brackets denote ensemble averaging. These balances are defined as follows

$$ {\text{MKE:}}\quad \matrix{  {\frac{D}{{Dt}}K + \frac{\partial }{{\partial {x_{j}}}}\left( {\left\langle {{U_{i}}} \right\rangle \left\langle {{u_{i}}{u_{j}}} \right\rangle + \frac{1}{{{\rho _{0}}}}\left\langle p \right\rangle \left\langle {{U_{j}}} \right\rangle - {\nu _{m}}\frac{\partial }{{\partial {x_{j}}}}K} \right) + } \hfill \\ { - \left\langle {{u_{i}}{u_{j}}} \right\rangle \frac{{\partial \left\langle {{U_{i}}} \right\rangle }}{{\partial {x_{j}}}} - \frac{{{g_{i}}\left\langle \rho \right\rangle }}{{{\rho _{0}}}}\left\langle {{U_{i}}} \right\rangle {\delta _{ {ij}}} + {\nu _{m}}{{\left( {\frac{{\partial \left\langle {{U_{j}}} \right\rangle }}{{\partial {x_{j}}}}} \right)}^{2}} = 0} \hfill \\ }<!end array> $$

$$ {\text{MPE}}:\quad \matrix{  {\frac{D}{{Dt}}\left( {\frac{1}{2}{{\left\langle \rho \right\rangle }^{2}}} \right) + \frac{\partial }{{\partial {x_{j}}}}\left( {\left\langle \rho \right\rangle \left\langle {\rho {u_{j}}} \right\rangle - {\kappa _{m}}\frac{\partial }{{\partial {x_{j}}}}\left( {\frac{1}{2}{{\left\langle \rho \right\rangle }^{2}}} \right)} \right) + } \hfill \\ { - \left\langle {\rho {u_{j}}} \right\rangle \frac{{\partial \left\langle \rho \right\rangle }}{{\partial {x_{j}}}} + {\kappa _{m}}{{\left( {\frac{{\partial \left\langle \rho \right\rangle }}{{\partial {x_{j}}}}} \right)}^{2}} = 0} \hfill \\ }<!end array> $$

In both balances, the first term denotes the material derivative K = 1/2·<*U*_*i*_>^2^, the second, third and fourth terms denote diffusive transport of kinetic energy by Reynolds stresses, pressure, and molecular viscosity (*υ*_*m*_), and diffusive transport of potential energy by buoyancy fluxes and molecular diffusivity (*κ*_*m*_), the fifth terms denote energy transfer by Reynolds stresses and buoyancy fluxes, the sixth term denote dissipation by molecular viscosity and diffusivity, and the seventh term transfer by gravity velocity correlations. Note that the advection term in the MPE balance also includes the latter term. Hence, this term can be considered to convert MKE to MPE and vice versa. Further note that the molecular terms contribute little to the loss of energy in the energy balances of the mean flow compared to the transfer of energy by the Reynolds stress and buoyancy flux. The turbulent fluxes transfer energy from the mean flow energy balances to the energy balances for the turbulent flow.

The turbulent kinetic and potential energy balances can be obtained by subtracting the RANS conservation equations for momentum and mass from the NS equations and subsequently multiplying the fluctuating balances with *u*_*i*_ and *ρ* and ensemble averaging the results. This procedure provides the following balance equations.

$$ {\text{TKE}}:\quad \matrix{  {\frac{D}{{Dt}}k + \frac{\partial }{{\partial {x_{j}}}}\left\langle {{u_{j}}k} \right\rangle + \frac{1}{{{\rho _{0}}}}\frac{\partial }{{\partial {x_{j}}}}\left\langle {p{u_{j}}} \right\rangle - {\nu _{m}}\frac{{{\partial ^{2}}}}{{\partial x_{j}^{2}}}k + } \hfill \\ { + \left\langle {{u_{i}}{u_{j}}} \right\rangle \frac{{\partial \left\langle {{U_{i}}} \right\rangle }}{{\partial {x_{j}}}} + {\nu _{m}}{{\left( {\frac{{\partial k}}{{\partial {x_{j}}}}} \right)}^{2}} + \frac{{{g_{3}}}}{{{\rho _{0}}}}\left\langle {\rho {u_{i}}} \right\rangle {\delta _{ {ij}}} = 0} \hfill \\ }<!end array> $$

$$ {\text{PE}}:\quad \matrix{  {\frac{D}{{Dt}}\left( {\frac{1}{2}\left\langle {{\rho ^{2}}} \right\rangle } \right) + \frac{\partial }{{\partial {x_{j}}}}\left( {\frac{1}{2}\left\langle {{\rho ^{2}}{u_{j}}} \right\rangle - {\kappa _{m}}\frac{{{\partial ^{2}}}}{{\partial x_{j}^{2}}}\left( {\frac{1}{2}\left\langle {{\rho ^{2}}} \right\rangle } \right)} \right) + } \hfill \\ { + \left\langle {\rho {u_{j}}} \right\rangle \frac{{\partial \left\langle \rho \right\rangle }}{{\partial {x_{j}}}} + {\kappa _{m}}{{\left( {\frac{{\partial \left\langle \rho \right\rangle }}{{\partial {x_{j}}}}} \right)}^{2}} = 0} \hfill \\ }<!end array> $$

In both balances, the first term is the material derivative, the second, third, and fourth terms are diffusive transport by velocity and pressure fluctuations and molecular viscosity or diffusivity. The fifth term turbulent kinetic (KE) and potential (PE) energy transfer between the mean flow and the turbulent flow by Reynolds stress and shear and buoyancy fluxes and density gradients, respectively. The sixth term represents dissipation by molecular viscosity (KE) and diffusivity (PE). The seventh term in the KE balance represents the buoyancy flux. Note that in these balances the molecular terms must be retained because velocity and density differences vary over smaller scales than compared to the mean flow balances. Hence, transfer of energy from the mean flow to the turbulent flow will be eventually dissipated by molecular viscosity and diffusivity.
